# Women’s ability to negotiate safer sex with partners by contraceptive status among a nationally representative sample of married women in Nigeria

**DOI:** 10.1186/s40834-023-00214-2

**Published:** 2023-03-01

**Authors:** Bola Lukman Solanke, Joseph Ayodeji Kupoluyi, Abayomi Folorunso Awoleye, Olusola Esther Adewole, Oyeyemi Bukola Babalola

**Affiliations:** 1 Department of Demography and Social Statistics, Obafemi Awolowo University, Ile-Ife, Nigeria; 2 Department of Sociology and Anthropology, Obafemi Awolowo University, Ile-Ife, Nigeria; 3 Department of Psychology, Obafemi Awolowo University, Ile-Ife, Nigeria

**Keywords:** Safer sex negotiation, Contraceptive users, Non-users, Sexual and reproductive health, Women, Nigeria

## Abstract

**Background:**

Existing studies established that safer sex negotiation influences contraceptive use, and women who are able to negotiate safer sex were expected to be contraceptive users. However, it is not certain that all contraceptive users have the ability to negotiate safer sex. Likewise, there is no evidence that all non-users are not able to negotiate safer sex with partners. The study assesses the prevalence of women’s ability to negotiate safer sex and examines the determinants of women’s ability to negotiate safer sex among contraceptive users and non-users.

**Methods:**

The comparative cross-sectional research design was adopted. Data were extracted from the 2018 Nigeria Demographic and Health Survey. The study analyzed a sample of 2,765 contraceptive users and 20,304 non-users. The outcome variable was women’s ability to negotiate safer sex with partners. The explanatory variables examined are eight socio-demographic characteristics (age, child marriage, education, parity, media exposure, religion, work status, and experience of female genital mutilation), six relational characteristics (healthcare autonomy, financial autonomy, household wealth quintile, partners’ education, ownership of assets, and type of marriage). Attitude to wife-beating, male controlling behavior, place of residence, and geo-political zone of residence were included as control variables. Multivariable regression models were estimated.

**Results:**

Findings showed that 6.2% of women who were not able to negotiate safer sex were contraceptive users, while 15.9% of women who were able to negotiate safer sex were contraceptive users. Among non-users, the significant determinants were child marriage, education, parity, mass media exposure, religion, work status, healthcare autonomy, financial autonomy, household wealth, partner education, type of marriage, geo-political zone, attitude to wife-beating, and male controlling behavior. Regarding contraceptive users, the significant determinants were parity, religion, the experience of female genital mutilation, financial autonomy, partner education, type of marriage, and the geo-political zone of residence.

**Conclusion:**

The ability to negotiate safer sex differs among contraceptive users and non-users. Also, the determinants of the ability to negotiate safer sex differ among contraceptive users and non-users. While existing strategies may continue to focus on women not using contraceptives, new strategies promoting reproductive autonomy are required among contraceptive users.

## Plain English Summary

Existing studies established that safer sex negotiation influences contraceptive use, and women who are able to negotiate safer sex were expected to be contraceptive users. However, it is not certain that all contraceptive users have the ability to negotiate safer sex. Likewise, there is no evidence that all non-users are not able to negotiate safer sex with partners. The objectives of the study were to assess the prevalence of women’s ability to negotiate safer sex and to examine the determinants of women’s ability to negotiate safer sex among contraceptive users and non-users. The comparative cross-sectional research design was adopted. Data were extracted from the most recent Nigeria Demographic and Health Survey. Samples of contraceptive users and non-users were analyzed in the study. The outcome variable was women’s ability to negotiate safer sex with partners. The explanatory variables examined are eight socio-demographic characteristics (age, child marriage, education, parity, media exposure, religion, work status, and experience of female genital mutilation), six relational characteristics (healthcare autonomy, financial autonomy, household wealth quintile, partners’ education, ownership of assets, and type of marriage). Attitude to wife-beating, male controlling behavior, place of residence, and geo-political zone of residence were included as control variables. Findings showed a higher ability to negotiate safer sex among contraceptive users. There were differences in the determinants of safer sex negotiation among contraceptive users and non-users. The study concluded that the ability to negotiate safer sex and its determinants differs among contraceptive users and non-users. It was suggested that while existing strategies may continue to focus on women not using contraceptives, new strategies promoting reproductive autonomy are required among contraceptive users.

## Background

Women’s ability to negotiate safer sex with partners refers to women’s capacity to refuse sex when not desired, and to request, partners to use a condom during intercourse [1, 2]. Studies in Nigeria [3–6] as well as in other countries [7, 8] have focused on safer sex negotiation with a view to improving women’s sexual and reproductive health autonomy. Women’s ability to negotiate safer sex with partners reflects three important development and public health concerns. One, it reflects gender norms within sexual or marital relationships. Across developing countries, marital norms and practices are tilted against women, and gender dynamics in many sub-Saharan African households undermine women’s reproductive health [9]. In many parts of Nigeria, women’s sexual health and rights are not well-recognized and respected, and men have the final say on women’s sexual and reproductive choices [10–13]. Similar harmful practices against women’s sexual and reproductive health have been documented in many other developing countries [14–17]. These practices greatly undermine women’s general well-being and also slow down the process of achieving gender equality in developing countries.

Two, it indicates the extent to which a woman is able to protect herself from sexually transmitted infections, unintended pregnancies, and the accompanying health and childbearing challenges. Many studies have documented how women’s lack of power to negotiate safer sex elevates their risks of sexually transmitted infections including HIV/AIDS, as well as unintended pregnancies, and abortion [18–22]. A recent study in Sierra Leone [23] observed that the rate of unintended pregnancies will continue to increase if women do not take the initiative to protect themselves. Two Nigerian studies [24, 25] particularly reported that ever-married women were more likely to be infected with HIV compared to unmarried women in the country. This is probably a result of the inability to request the use of a condom by the partners of the studied women. Studies have also linked a lack of autonomy in sexual relations to other adverse health outcomes such as mental health challenges [26], and newborn and infant morbidity [27]. Three, it also reflects the level of women’s empowerment in society, which numerous studies [28–34] have found to strongly impact contraceptive use, and the ability to negotiate safer sex [35]. It is reasoned that through empowerment, women’s autonomy and agency improve. This equally improves their ability to negotiate safer sex and ultimately leads to contraceptive use. By this logic, safer sex negotiation influences contraceptive use, and women who are able to negotiate safer sex were expected to be contraceptive users.

However, it is not certain that all contraceptive users have the ability to negotiate safer sex with partners. For instance, most women who are using modern contraceptives covertly most probably do so due to their inability to negotiate with partners or inability to get partners’ approval for the desired contraceptive method [36–38]. Likewise, there is no evidence that all non-users are not able to negotiate safer sex with partners since some women are non-users due to health concerns or other social reasons [39, 40]. These realities have thus created the need to ascertain whether women’s ability to negotiate safer sex differs by their contraceptive status. This information is important for the purpose of designing safer sex negotiation strategies that resonate with women’s contraceptive status. The study was guided by two research questions, namely, what is the prevalence of women’s ability to negotiate safer sex with partners among contraceptive users and non-users, and do the determinants of women’s ability to negotiate safer sex with partners differ among contraceptive users and non-users? Findings will inform the 2021 National Policy on Population for Sustainable Development [41]. The policy targets wide-spectrum population-related issues, including gender equality, women empowerment, and male involvement in reproductive health as means to accelerate the attainment of sustainable growth and development in the country. However, the implementation strategies could be strengthened to accommodate more measures to promote women’s sexual and reproductive health, particularly at the household level.

## Methods

### Study design

The comparative cross-sectional research design was adopted in the study. This design was suitable for the study since the study goal was to examine women’s ability to negotiate safer sex and its determinants by contraceptive status. The design proceeds by first computing the prevalence of women’s ability to negotiate safer sex with partners for the two groups of women, namely, users and non-users. The Stata prtest command [42] was then performed to test for differences in the proportions of women using or not using modern contraceptives. The rationale for this test was to determine whether the two groups are the same or not. The preliminary results revealed significant differences between the two groups, which suggests that the groups are independent and require separate analyses. Secondly, regression models were fitted separately for contraceptive users and non-users using the same explanatory variables, and the results were compared.

### Data source

This study analyzed data extracted from the 2018 Nigeria Demographic and Health Survey (NDHS). The 2018 NDHS was the seventh round of the Demographic and Health Survey (DHS) implemented in Nigeria. The survey was executed by the National Population Commission (NPC) in collaboration with the National Malaria Elimination Programme (NMEP) [43]. Financial and technical support for the execution of the survey was provided by a number of international development partners such as the Global Fund, Bill and Melinda Gates Foundation (BMGF), United States Agency for International Development (USAID), and the United Nations Population Fund (UNFPA). The importance of the 2018 NDHS was the provision of reliable estimates of national demographic and health characteristics, which is relevant for tracking the attainment of the Sustainable Development Goals (SDGs) in the country [43].

### Sampling

The NPC and ICF has published comprehensive detail of the survey methodology adopted for the 2018 NDHS. This is widely available via https://dhsprogram.com/pubs/pdf/FR359/FR359.pdf. It is however important to provide a brief as it relates to the current study. A multi-stage sampling procedure was employed in the survey. In the first stage, the country was stratified into urban and rural areas. Using a two-stage procedure, primary sampling units were first selected. This was followed by the selection of households for the study through systematic random sampling. Men and women were then randomly recruited for interviews in the selected households. Overall, 41,821 women and 13,311 men were completely interviewed in the survey [43].

### Participants

This study was based on the women’s data which covered 41,821 women. However, women who were never married (10,669) and those not sexually active (8,043) in the last four weeks preceding the survey were excluded from the study. The reason for their exclusion was to ensure that included women were either in marital or sexual relationships. Though many unmarried women are also sexually active, they are not included in the study due to the absence of a regular partner whose characteristics are examined in the study. The study thus analyzed a sample of 23,109 women. The sample was divided based on women’s current contraceptive status, namely, the user group, and the non-user group. The user group comprised 2,765 women, while the non-user group comprised 20,344 women. The DHS weighting factors were applied to weight the sample.

### Outcome variable

The outcome variable in the study was women’s ability to negotiate safer sex with partners. This was measured by women’s responses to whether they can refuse sex with partners and whether they can ask their partners to use a condom during intercourse. A composite index dividing the responses into two categories of ‘able to negotiate safer sex’ and ‘not able to negotiate safer sex’ was then generated. This measure has been widely used for safer sex negotiation in many existing studies [1, 2, 4]. The category of interest in the study was the group of women who are able to negotiate safer sex.

### Explanatory variables

Three sets of explanatory variables were selected based on the findings of existing empirical studies. One, eight socio-demographic characteristics associated with safer sex negotiation in previous studies were selected. These are age [3], child marriage [44], education [32], parity [45], media exposure [1], religion [4], work status [2], and experience of female genital mutilation [46]. Age was grouped into three categories 15–24, 25–34, and 35 years or older. Child marriage was measured as ‘yes’ if the age at first marriage was less than eighteen years, and ‘no’ if otherwise. Parity was grouped as ‘nulliparity’ if women have had no previous live birth, ‘primiparity’ if women have had only a child, ‘multiparity’ if women have had between two to four live births, and ‘grand multiparity’ if women have had five or more live births. This is in line with the categorization of women’s parity in the literature [47–49]. Media exposure was derived from the frequencies of reading newspapers, listening to the radio, and watching television through the generation of a composite index. The index was divided into three parts to reflect low, moderate, and high media exposure.

Two, six relational characteristics were selected based on findings in relevant existing studies. These are healthcare autonomy [50], financial autonomy [51], household wealth quintile [32], partners’ education [32], ownership of assets [32], and type of marriage [52]. Healthcare autonomy was based on who had the final say on women’s healthcare, while financial autonomy was based on who had the final say on spending women’s earnings. In both cases, women had autonomy if they had the final say either solely or jointly with partners. Ownership of assets was based on ownership of land/house either solely or jointly with a partner. Three, two gender norms, namely, attitude to wife-beating and male controlling behavior, and two societal characteristics, namely place of residence and geo-political zone of residence were included as control variables in the study. Attitude to wife-beating was grouped as ‘supportive’ if women accepted any condition for wife-beating, and non-supportive if wife-beating was rejected given all conditions. Male controlling behavior was derived from women’s responses to whether the husband or partner desire to limit the wife’s contact, desire to know her movement, gives no permission to meet friends, alleges unfaithfulness, or is jealous of the wife’s interaction with other men. The four control variables have been found to be correlates of safer sex negotiation or other women’s sexual outcomes in previous studies [2–4, 12, 32].

### Data analysis

Stata 14 [42] was used to perform data analyses. Respondents’ profile was described using frequency distribution and percentages. The prevalence of safer sex negotiation was described using a chart. A binary logistic regression analysis using unadjusted odds ratios was performed to select variables into multivariable logistic regression models based on either of two conditions. One, the variables to be included should show significance at *p* < 0.025. Two, variables to be included should have a variance inflation factor of less than ten. These conditions ensure that the regression model estimates are not misleading. Three multivariable regression models were estimated using the adjusted Odds ratio (aOR) with a 95% confidence interval. Model 1 included only the socio-demographic characteristics. Model 2 controlled for the relational characteristics, while Model 3 was the full model. The models were fitted separately for each group. Statistical significance was set at *p* < 0.05.

## Results

### Socio-demographic profile of respondents

Table 1 presents the socio-demographic profile of the respondents by their contraceptive status. In both groups, women aged 25 years or older were dominant in the samples compared to young adults. More than half (57.4%) of non-users had early marriage compared to slightly above one-third (35.2%) of respondents who were contraceptive users. The proportion of women without formal education was higher (51.2%) among non-users compared to the proportion among users (15.0%). Also, the proportions of respondents at each level of educational attainment were consistently higher among contraceptive users compared to non-users. The proportions of nulliparous and primiparous women were higher among non-users while the proportions of multiparous and grand multiparous women were higher among contraceptive users. The distribution of respondents by mass media exposure showed that contraceptive users were better off. A higher proportion of Christian women (61.2%) were contraceptive users compared to the proportion of Muslim women (38.2%). The majority of respondents were employed but the proportion of employed women was higher among users compared to non-users. The experiences of female genital mutilation were slightly higher among contraceptive users.

**Table 1:** Socio-demographic profile of respondents by contraceptive status

Characteristic	Non-users	Users
	Number (%)	Number (%)
**Age group (years)**		
15-24	4,849 (23.8)	295 (10.7)
25-34	8,007 (39.4)	1,199 (43.4)
35 or older	7,488 (36.8)	1,270 (45.9
**Child marriage**		
Yes	11,676 (57.4)	973 (35.2)
No	8,668 (43.6)	1,792 (64.8)
**Education**		
None	10,419 (51.2)	515 (15.0)
Primary	2,881 (14.2)	514 (18.6)
Secondary	5,327 (26.2)	1,320 (47.4)
Higher	1,717 (8.4)	516 (18.7)
**Parity**		
Nulliparity	1,753 (8.6)	24 (0.9)
Primiparity	5,806 (28.5)	704 (25.5)
Multiparity	5,154 (25.3)	992 (35.9)
Grand multiparity	7,631 (37.5)	1,045 (38.8)
**Mass media exposure**		
Low	8,215 (40.4)	449 (16.2)
Moderate	8,545 (42.0)	1,366 (49.4)
High	3,584 (17.6)	950 (34.4)
**Religion**		
Christianity	6,744 (33.2)	1,692 (61.2)
Islam	13,491 (66.3)	1,067 (38.2)
Others	110 (0.5)	06 (0.2)
**Work status**		
Unemployed	6,494 (31.9)	459 (16.6)
Employed	13,851 (68.1)	2,306 (83.4)
**Experienced female genital mutilation**		
No	17,814 (87.6)	2,346 (84.9)
Yes	2,530 (12.4)	419 (15.1)
**Healthcare autonomy**		
No	12,580 (61.8)	1,071 (38.7)
Yes	7,764 (38.2)	1,694 (61.3)
**Financial autonomy**		
No	9,245 (45.4)	931 (33.7)
Yes	11,099 (54.6)	1,834 (66.3)
**Total**	20,344 (100.0)	2,765 (100.0)
**Household wealth**		
Poorest	4,830 (23.7)	163 (5.9)
Poorer	4,756 (23.4)	319 (11.5)
Middle	3,856 (19.0)	494 (17.9)
Richer	3,488 (17.1)	791 (28.6)
Richest	3,414 (16.8)	997 (36.1)
**Ownership of assets**		
Does not own	17,058 (83.8)	2,125 (76.9)
Owned assets	3,286 (16.2)	640 (23.1)
**Partners’ education**		
None	8,675 (42.6)	366 (13.2)
Primary	2,758 (13.6)	342 (12.4)
Secondary	6,073 (29.8)	1,279 (46.3)
Higher	2,838 (14.0)	77.8 (28.1)
**Type of marriage**		
Monogamy	13,451 (66.1)	2,178 (78.8)
Polygyny	6,893 (33.9)	587 (21.2)
**Place of residence**		
Urban	7,355 (36.1)	1,686 (60.9)
Rural	12,989 (63.9)	1,079 (39.1)
**Geo-political zone**		
North-central	2,485 (12.2)	482 (17.4)
North-east	3,927 (19.3)	264 (9.6)
North-west	8,012 (39.4)	568 (20.5)
South-east	1,590 (7.8)	236 (8.5)
South-south	1,753 (8.6)	371 (13.4)
South-west	2,576 (12.7)	843 (30.5)
**Attitude to wife-beating**		
Not supportive	13,464 (66.2)	2,278 (82.4)
Supportive	6,880 (33.8)	487 (17.6)
**Male controlling behaviour**		
Low	18,302 (89.9)	2,440 (88.3)
Moderate	1,636 (8.1)	256 (9.2)
High	406 (2.0)	69 (2.5)
**Total**	20,344 (100.0)	2,765 (100.0)

Source: Authors’ analysis based on 2018 Nigeria Demographic and Health Survey

In terms of autonomy, more contraceptive users had both healthcare and financial autonomy than non-users. Women in the poorest and poorer household wealth groups were dominant among non-users, while women in the richer and richest household wealth groups were dominant among contraceptive users. Respondents’ distribution by partners’ education revealed that the partners of non-users were worst off compared to the partners of contraceptive users. Though, ownership of assets was poor among respondents but the proportion of women who owned assets was higher among users compared to non-users (23.1% vs. 16.2%). The majority of both users and non-users were in monogamous marriages. Rural dwellers were dominant among non-users, while urban residents were dominant among contraceptive users. The highest proportion of contraceptive users was domiciled in the Southwest zone, while the highest proportion of non-users was domiciled in the Northwest zone. The majority of women were not supportive of wife-beating. However, more than one-third (33.8%) of non-users were supportive of wife-beating compared to the proportion among users (17.6%). The majority of women in both groups reported a low degree of male controlling behavior.

### Prevalence of women’s ability to negotiate safer sex among contraceptive users and non-users

**Fig. 1:**
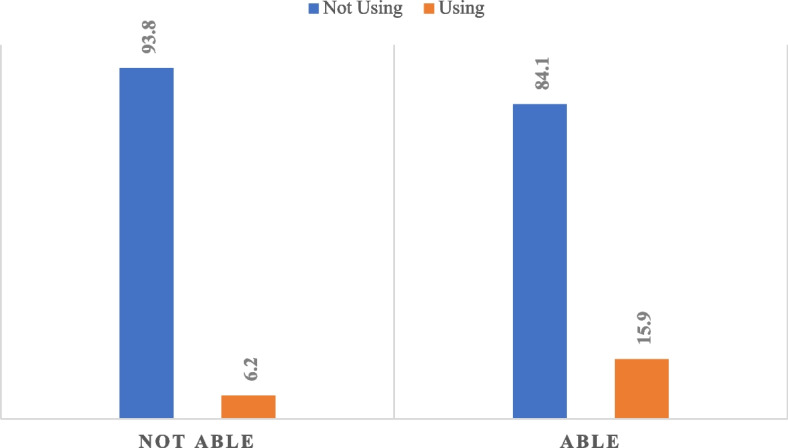
Women’s ability to negotiate safer sex by contraceptive status

Figure 1 presents the proportions of women who are able or not able to negotiate safer sex by their contraceptive status. As shown in the figure, the majority (93.8%) of women who were not able to negotiate safer sex were equally non-users of contraceptives. However, 6.2% of them were current contraceptive users. In contrast, among women who were able to negotiate safer sex with partners, the majority (84.1%) were not current contraceptives users, while a higher proportion (15.9%) compared to the other group of women were current contraceptive users.

### Determinants of women’s ability to negotiate safer sex among non-users of contraceptives

Table 2 presents the influence of the socio-demographic characteristics on the ability to negotiate safer sex among non-users. In Model 1, age, working status, and experience of female genital mutilation showed no statistical significance. Child marriage, education, parity, mass media exposure, and religion had significant effects on women’s ability to negotiate safe sex. With the inclusion of the relational characteristics in Model 2, age and experience of female genital mutilation remained insignificant but working status became strengthened. Other variables maintained their significance in Model 2 as revealed in Model 1. In the model, only ownership of assets lacked statistical significance. Other variables in the model had significant effects on women’s ability to negotiate safer sex. In Model 3, age, and experience of female genital mutilation were the individual characteristics with no significant influence on the ability to negotiate safer sex, while ownership of assets was the only insignificant relational characteristic.

**Table 2:** Effects of socio-demographic characteristics on ability to negotiate safer sex among non-users

Characteristic predicting ability to negotiate safer sex	Model 1		Model 2		Model 3	
	aOR	95% CI	aOR	95% CI	aOR	95% CI
**Age group (years)**						
15-24 ^RC^	1.000	-	1.000	-	1.000	-
25-34	0.945	0.843-1.058	0.945	0.838-1.064	0.922	0.818-1.040
35 or older	0.890	0.752-1.053	0.887	0.743-1.058	0.858	0.723-1.018
**Child marriage**						
Yes ^RC^	1.000	-	1.000	-	1.000	-
No	1.302**	1.193-1.420	1.209**	1.106-1.321	1.169**	1.070-1.278
**Education**						
None ^RC^	1.000	-	1.000	-	1.000	-
Primary	1.504**	1.331-1.700	1.129	0.999-1.276	1.120	0.991-1.265
Secondary	2.048**	1.807-2.320	1.312**	1.144-1.506	1.313**	1.139-1.513
Higher	3.056**	2.460-3.797	1.638**	1.283-2.090	1.582**	1.232-2.030
**Parity**						
Nulliparity ^RC^	1.000	-	1.000	-	1.000	-
Primiparity	1.162*	1.002-1.347	1.137	0.980-1.318	1.124	0.966-1.308
Multiparity	1.288*	1.105-1.502	1.258*	1.079-1.466	1.255*	1.073-1.468
Grand multiparity	1.289*	1.081-1.535	1.298*	1.085-1.552	1.329*	1.108-1.594
**Mass media exposure**						
Low ^RC^	1.000	-	1.000	-	1.000	-
Moderate	1.250**	1.108-1.409	1.055	0.932-1.195	1.098	0.969-1.245
High	1.607**	1.359-1.899	1.225*	1.025-1.464	1.276*	1.064-1.529
**Religion**						
Christianity ^RC^	1.000	-	1.000	-	1.000	-
Islam	0.408**	0.361-0.461	0.455**	0.400-0.518	0.479**	0.412-0.557
Others	0.521*	0.287-0.946	0.675	0.393-1.157	0.801	0.489-1.323
**Work status**						
Unemployed ^RC^	1.000	-	1.000	-	1.000	-
Employed	0.994	0.883-1.119	0.717**	0.623-0.825	0.697**	0.603-0.805
**Experienced female genital mutilation**						
No ^RC^	1.000	-	1.000	-	1.000	-
Yes	1.100	0.958-1.262	1.088	0.942-1.256	1.130	0.973-1.313
**Healthcare autonomy**						
No ^RC^			1.000	-	1.000	-
Yes			1.396**	1.230-1.585	1.313**	1.159-1.488
**Financial autonomy**						
No ^RC^			1.000	-	1.000	-
Yes			1.515**	1.338-1.715	1.525**	1.345-1.729
**Household wealth**						
Poorest ^RC^			1.000	-	1.000	-
Poorer			0.961	0.838-1.106	0.993	0.866-1.139
Middle			1.199*	1.009-1.425	1.194	0.999-1.426
Richer			1.294*	1.086-1.541	1.248*	1.034-1.506
Richest			1.293*	1.039-1.602	1.262	0.986-1.616
**Partners’ education**						
None ^RC^			1.000	-	1.000	-
Primary			1.393**	1.211-1.602	1.431**	1.245-1.644
Secondary			1.575**	1.375-1.804	1.585**	1.387-1.811
Higher			1.845**	1.530-2.256	1.834**	1.516-2.220
**Ownership of assets**						
Does not own ^RC^			1.000	-	1.000	-
Owned assets			1.076	0.958-1.208	1.026	0.916-1.150
**Type of marriage**						
Monogamy ^RC^			1.000	-	1.000	-
Polygyny			0.731**	0.666-0.802	0.736**	0.670-0.808
**Place of residence**						
Urban ^RC^					1.000	-
Rural					0.947	0.816-1.098
**Geo-political zone**						
North-central ^RC^					1.000	-
North-east					1.453**	1.207-1.748
North-west					0.723**	0.604-0.866
South-east					0.896	0.688-1.166
South-south					0.995	0.809-1.223
South-west					0.791	0.630-0.993
**Attitude to wife-beating**						
Not supportive ^RC^					1.000	-
Supportive					0.656**	0.584-0.737
**Male controlling behaviour**						
Low ^RC^					1.000	-
Moderate					0.868*	0.756-0.997
High					0.823	0.649-1.043

*aOR* adjusted Odds Ratio, *CI* Confidence interval, *p<0.05, **p<0.01

^RC^Reference category

In the model, women who did not have child marriage were more likely to negotiate safer sex with partners compared to women who had child marriage (aOR = 1.169; 95% CI: 1.070–1.278). The odds of women’s ability to negotiate safer sex increased significantly and consistently as educational attainments increased. Multiparous (aOR = 1.255; 95% CI: 1.073–1.468) and grand multiparous women (aOR = 1.329; 95% CI: 1.108–1.594) had a higher likelihood of negotiating safer sex compared to nulliparous women. Women who had high media exposure were more likely to negotiate safer sex compared to women who had low media exposure (aOR = 1.276; 95% CI: 1.064–1.529). Muslim women compared to Christian women were less likely to negotiate safer sex (aOR = 0.479; 95% CI: 0.412–0.557). Likewise, employed women had lower odds of negotiating safer sex.

Women who had both healthcare and financial autonomy were more likely to negotiate safer sex. Only women in the richer households had higher significant odds of negotiating safer sex (aOR = 1.248; 95% CI: 1.034–1.506). The odds of negotiating safer sex increased consistently as partners’ education improves. Women in polygynous unions were less likely to negotiate safer sex compared to monogamous women (aOR = 0.736; 95% CI: 0.670–0.808). While women in the Northeast zone had more likelihood of negotiating safer sex, women in the Northwest and Southwest zones had less likelihood of negotiating safer sex. Women who supported wife-beating were less likely to negotiate safer sex (aOR = 0.656; 95% CI: 0.584–0.737). Similarly, women who had moderate media exposure were less likely to negotiate safer sex (aOR = 0.868; 95% CI: 0.756–0.997).

### Determinants of women’s ability to negotiate safer sex among contraceptive users

Table 3 presents the effects of the socio-demographic characteristics on the ability to negotiate safer sex among contraceptive users. In Model 1, most of the variables included did not reveal any significant effect. Nevertheless, higher education showed a significant influence on safer sex negotiation. Also, religion and experience of female genital mutilation had a significant influence on women’s ability to negotiate safer sex. In Model 2, age, child marriage, education, mass media exposure, and work status remained without statistical significance, while parity and religion had significant effects on the ability to negotiate safer sex. The experience of female genital mutilation which was strong in Model 1 was weakened in Model 2 by the inclusion of the relational characteristics in the model. Three of the relational characteristics, namely, financial autonomy, partners’ education, and type of marriage showed significant effects, while the three other characteristics, namely, healthcare autonomy, household wealth, and ownership of assets were insignificant in their effects on safer sex negotiation.

**Table 3:** Effects of socio-demographic characteristics on ability to negotiate safer sex among contraceptive users

Characteristic predicting ability to negotiate safer sex	Model 1		Model 2		Model 3	
	aOR	95% CI	aOR	95% CI	aOR	95% CI
**Age group (years)**						
15-24 ^RC^	1.000	-	1.000	-	1.000	-
25-34	0.759	0.499-1.154	0.762	0.497-1.167	0.759	0.493-1.168
35 or older	0.681	0.409-1.133	0.676	0.392-1.167	0.682	0.404-1.151
**Child marriage**						
Yes ^RC^	1.000	-	1.000	-	1.000	-
No	1.298	0.983-1.713	1.192	0.869-1.634	1.234	0.903-1.686
**Education**						
None ^RC^	1.000	-	1.000	-	1.000	-
Primary	1.065	0.692-1.637	0.727	0.422-1.252	0.752	0.442-1.280
Secondary	1.346	0.919-1.972	0.799	0.475-1.344	0.823	0.496-1.364
Higher	1.905*	1.171-3.099	1.004	0.557-1.812	0.998	0.578-1.786
**Parity**						
Nulliparity ^RC^	1.000	-	1.000	-	1.000	-
Primiparity	0.402	0.121-1.338	0.343*	0.135-0.877	0.338*	0.133-0.856
Multiparity	0.458	0.137-1.533	0.375*	0.143-0.979	0.367*	0.140-0.963
Grand multiparity	0.538	0.154-1.881	0.471	0.167-1.332	0.450	0.159-1.271
**Mass media exposure**						
Low ^RC^	1.000	-	1.000	-	1.000	-
Moderate	1.292	0.898-1.858	1.065	0.676-1.676	1.167	0.742-1.836
High	1.408	0.926-2.140	1.136	0.691-1.869	1.240	0.756-2.035
**Religion**						
Christianity ^RC^	1.000	-	1.000	-	1.000	-
Islam	0.468**	0.368-0.594	0.497**	0.387-0.640	0.411**	0.309-0.548
Others	0.234*	0.067-0.811	0.329*	0.113-0.956	0.388	0.133-1.133
**Work status**						
Unemployed ^RC^	1.000	-	1.000	-	1.000	-
Employed	1.053	0.752-1.475	0.740	0.468-1.169	0.738	0.463-1.175
**Experienced female genital mutilation**						
No ^RC^	1.000	-	1.000	-	1.000	-
Yes	1.535*	1.013-2.326	1.499	0.966-2.326	1.652*	1.029-2.652
**Healthcare autonomy**						
No ^RC^			1.000	-	1.000	-
Yes			1.195	0.888-1.608	1.234	0.922-1.652
**Financial autonomy**						
No ^RC^			1.000	-	1.000	-
Yes			1.881**	1.376-2.572	1.920*	1.388-2.655
**Household wealth**						
Poorest ^RC^			1.000	-	1.000	-
Poorer			0.913	0.540-1.543	0.951	0.560-1.617
Middle			0.955	0.536-1.703	1.117	0.630-1.980
Richer			0.780	0.430-1.414	0.929	0.500-1.726
Richest			1.111	0.598-2.066	1.392	0.727-2.667
**Partners’ education**						
None ^RC^			1.000	-	1.000	-
Primary			2.737**	1.598-4.688	2.822**	1.640-4.858
Secondary			3.498**	2.122-5.769	3.381**	2.060-5.550
Higher			2.810**	1.683-4.692	2.509**	1.508-4.174
**Ownership of assets**						
Does not own ^RC^			1.000	-	1.000	-
Owned assets			0.987	0.731-1.333	1.003	0.750-1.342
**Type of marriage**						
Monogamy ^RC^			1.000	-	1.000	-
Polygyny			0.597**	0.458-0.779	0.584**	0.448-0.762
**Place of residence**						
Urban ^RC^					1.000	-
Rural					0.962	0.690-1.341
**Geo-political zone**						
North-central ^RC^					1.000	-
North-east					1.991*	1.205-3.290
North-west					1.131	0.764-1.674
South-east					0.639	0.370-1.105
South-south					0.726	0.497-1.061
South-west					0.895	0.605-1.324
**Attitude to wife-beating**						
Not supportive ^RC^					1.000	-
Supportive					0.971	0.655-1.439
**Male controlling behaviour**						
Low ^RC^					1.000	-
Moderate					1.201	0.781-1.848
High					0.861	0.495-1.497

*aOR* adjusted Odds Ratio, *CI* Confidence interval, *p<0.05, **p<0.01

^RC^Reference category

In Model 3, three individual characteristics, namely, parity, religion, and experience of female genital mutilation revealed significant effects. Primiparous (aOR = 0.338; 95% CI: 0.133–0.856) and multiparous (aOR = 0.367; 95% CI: 0.140–0.963) women were less likely to negotiate safer sex. Likewise, Muslim women were less likely to negotiate safer sex compared to Christian women (aOR = 0.411; 95% CI: 0.309–0.548). In contrast, women who experienced female genital mutilation were more likely to negotiate safer sex (aOR = 1.652; 95% CI: 1.029–2.652). Also, three relational characteristics, namely, financial autonomy, partners’ education, and type of marriage revealed significant effects on the ability to negotiate safer sex. Women who had financial autonomy were nearly two times more likely to negotiate safer sex compared to women who had no autonomy (aOR = 1.920; 95% CI: 1.388–2.655). While the odds of safer sex negotiation increased significantly as partners’ education increased, the odds were lower among polygynous women compared to monogamous women (aOR = 0.584; 95% CI: 0.448–0.762). Only women in the Northeast had higher odds of negotiating safer sex compared to Northcentral women (aOR = 1.991; 95% CI: 1.205–3.290).

## Discussion

Improving women’s autonomy in sexual and reproductive health decision-making is central to the attainment of the sustainable development goal of gender equity and women empowerment. But in contemporary Nigeria, women’s autonomy in sexual and reproductive health matters is endangered by the persistence of patriarchy, which continues to promote some cultural norms and practices such as polygyny, child marriages, gender-based violence, son preference, and widow inheritance that subjugate women to the authority and control by men [41]. In some Nigerian communities, sexual abuse and violence, women’s lack of access to sexual and reproductive healthcare, and outright denial of women’s sexual rights are condoned by cultural beliefs and practices [10, 13, 53]. These lower the status of Nigerian women, and predispose many of them to adverse reproductive outcomes. As observed in some existing Nigerian studies [3, 11, 54], the lack of autonomy compromised women’s ability to negotiate safer sex and encourages inequality within unions.

The threshold of women’s autonomy is the ability to negotiate safer sex with partners. Such negotiation in many developing countries where patriarchy remained dominant is likely to lead to a positive change in existing gender norms within marital or sexual relationships, which may translate into improving women’s capacity to protect themselves from sexually transmitted infections [22, 25], unintended pregnancies [23], and other adverse health outcomes [26, 27]. In this paper, we assessed the prevalence of women’s ability to negotiate safer sex by contraceptive status, and also examined the determinants of women’s ability to negotiate safer sex among contraceptive users and non-users. This information has not been provided in existing studies on safer sex negotiation in Nigeria or elsewhere [1–4, 44, 46]. The findings in the study provide four additional pieces of information required to strengthen strategies for improving women’s sexual and reproductive health in Nigeria.

One, the finding showed that modern contraceptive usage remained low in Nigeria regardless of women’s ability to negotiate safer sex with partners. This has serious implications for the fertility level in the country by sustaining momentum for the growth of the population. Without improvement in the national contraceptive prevalence rate, it will be extremely difficult to achieve a demographic transition to a low fertility level in the country. As noted by the existing policy [41], the low modern contraceptive prevalence rate is one of the key factors sustaining high fertility in the country. Though a series of strategies are being implemented in the country to boost access to and utilization of family planning services, more strategies could still be developed for implementation, particularly at the family and household levels. For instance, the national population commission could develop a family planning education and communication program to be disseminated to new couples during solemnization at official marriage registries. The program should seek to promote the use of contraceptives as a joint responsibility of men and women in marital unions. This is important because many men as observed in a recent study [23] consider contraceptive use to be the responsibility of women.

Two, some women who were not able to negotiate safer sex with partners were contraceptive users. There is a high likelihood that this group of women was using contraceptives without the knowledge of their partners. As evident in previous studies [36–38], women may decide to covertly use contraception due to the inability to have a fruitful discussion with partners or partners’ disapproval of the method used. There are also cases where attempts to negotiate safer sex have led to incidences of spousal violence [6]. Rather than promote the covert use of contraceptives as a means of women’s autonomy, it is more important to take steps to address whatever reasons that could account for the covert use of contraceptives among women. This is because the discovery of covert use of contraceptives by an unsupportive partner may lead to marital disharmony and sometimes violence which does not in any way promote the institution of marriage. Since male disapproval is reported to be one of the main causes of the covert use of contraceptives by women [36–38], it is thus important that family planning managers in the country should give more relevance to more couple-oriented approaches to improving access to family planning services for married men and women in the country.

Three, women’s socio-demographic context matters in both safer sex negotiation and contraceptive use. As found in the study, women who are not able to negotiate safer sex were mostly non-users of contraceptives, and in terms of all the socio-demographic characteristics examined in the study, non-users were the worst off. This suggests that most contraceptive users may have attained some level of empowerment such as being more educated, being employed, or not being married as a child, which enhanced their contraceptive knowledge and use. This provides support for the higher contraceptive use found among empowered women in many existing studies [28–30, 33, 34]. It may also account for a higher ability to negotiate safer sex as reported in some studies [32, 35].

There is a possibility that empowerment and safer sex negotiation have a reverse causation. On one hand, women’s ability to negotiate safer sex may be a tool of empowerment. Through negotiation, women are able to reduce their risk of unintended pregnancies and childbearing, and infection with sexually transmitted diseases, which not only avail them more opportunities for economic productivity, and income generation, but may also translate to a higher level of equity within their marriages, especially in respect of sexual and reproductive health matters. In addition, the ability to negotiate safer sex enhance women’s self-esteem and self-efficacy, which has an important role to play in the process of attaining women’s empowerment [55]. On the other hand, women’s empowerment is able to improve both women’s ability to negotiate safer sex as well as contraceptive use because by exposure to better education, mass media, and women’s affirmative actions, the resultant change of attitude often promotes women’s autonomy in sexual and reproductive health decision-making. Thus, improving women’s sexual and reproductive autonomy in Nigeria requires that more attention be paid to women’s socio-demographic conditions. Strategies being implemented to improve women’s education, skills acquisition, access to credit facilities, and participation in the political process are pivotal. However, the strategies should be complemented by programs stressing the value of women’s autonomy in family and societal health.

Four, the determinants of women’s ability to negotiate safer sex differ among contraceptive users and non-users. In line with existing studies, it was found among non-users that child marriage [44], mass media [1], healthcare autonomy [50], financial autonomy [51], parity [45], household wealth, and partners’ education [4] are important determinants of safer sex negotiation. However, most of these determinants were not found significant among contraceptive users. The dominance of non-users in previous studies may have hidden this feature and may have misled family planning authorities to assume that the same set of factors predicts the ability to negotiate safer sex among all women. The separation of contraceptive users and non-users in this study has thus brought to the fore of sexual and reproductive health programming that a singular initiative may not work optimally and contraceptive users and non-users. The four determinants that cut across the two groups are religion, financial autonomy, partners’ education, and type of marriage. The policy implication of this finding is that many of the existing strategies to promote safer sex negotiation among women may work optimally among women not using contraceptives but not among those using contraceptives. This is because women using contraceptives have similar characteristics to women who have the ability to negotiate safer sex. While existing strategies may continue to focus on women not using contraceptives, new strategies are required for implementation among contraceptive users. Such programs should go beyond safer sex negotiation to promote reproductive autonomy, which not only embodies women’s autonomy on contraceptive use, pregnancy, and childbearing but also represent the future of women’s autonomy in sexual and reproductive health.

### Strength and limitations

To the best of our knowledge, no studies in Nigeria have examined whether women’s ability to negotiate safer sex differs by contraceptive status. By providing the information, this study made an original contribution to safer sex negotiation literature in Nigeria. However, the analysis performed in the study did not include a sensitivity analysis which may confirm the influence of safer sex negotiation on contraceptive use. Also, the cross-sectional data analyzed in the study connotes that in practical terms the use of the word ‘determinants’ in relation to safer sex negotiation does not necessarily imply effects but a significant correlation between the outcome and explanatory variables.

## Conclusion

The study ascertained that the prevalence of women’s ability to negotiate safer sex with partners differs among contraceptive users and non-users, and also confirmed that the determinants of women’s ability to negotiate safer sex with partners differ among contraceptive users and non-users. Findings imply that existing strategies to promote safer sex negotiation among women may work optimally among women not using contraceptives but not among those using contraceptives. We suggest that while existing strategies may continue to focus on women not using contraceptives, new strategies focusing on reproductive autonomy are required for implementation among contraceptive users.

## Data Availability

Data analysed in the study is available in the public domain. Interested researchers could access the dataset online at. https://dhsprogram.com/data/dataset/Nigeria_Standard-DHS_2018.cfm?flag=1.

## Abbreviations

aOR: Adjusted Odds Ratio
DHS: Demographic and Health Survey
NDHS: Nigeria Demographic and Health Survey
NPC: National Population Commission
